# Bacterial pathogens and antimicrobial susceptibility profiles in acute appendicitis in children: a retrospective study at a tertiary hospital in Ningbo, China

**DOI:** 10.3389/fped.2026.1871617

**Published:** 2026-07-17

**Authors:** Jingjuan Chen, Wenyuan Liu, Yibo Chen, Wenbo Lu, Yunlong Liu, Yefang Ke

**Affiliations:** 1 Department of Clinical Laboratory, Women and Children's Hospital of Ningbo University, Ningbo, Zhejiang, China; 2 Department of Pediatric Surgery, Women and Children’s Hospital of Ningbo University, Ningbo, Zhejiang, China

**Keywords:** acute appendicitis, antimicrobial resistance, bacteria, children, intraoperative specimens

## Abstract

**Objective:**

To investigate the distribution of bacterial pathogens in intraoperative specimens from children with acute appendicitis, characterize the antimicrobial resistance profiles of predominant Gram-negative bacteria, and compare microbiological differences between non-gangrenous and gangrenous appendicitis.

**Methods:**

We retrospectively reviewed records of patients aged below 18 years who underwent appendectomy for pathologically confirmed acute appendicitis at a tertiary hospital in Ningbo, China, between January 2019 and December 2024. Intraoperative pus, peritoneal fluid, or drainage fluid was cultured aerobically, and isolates were identified and tested for antimicrobial susceptibility. Pathology-stratified analyses compared infection type and major pathogen detection between non-gangrenous and gangrenous appendicitis. *Escherichia coli* resistance outcomes were additionally compared across age groups.

**Results:**

Among 1,981 children, 1,393 (70.31%) had positive cultures, yielding 1,903 isolates. Gram-negative organisms accounted for 81.14%. The three most common Gram-negative pathogens were *Escherichia coli* (55.12%), *Pseudomonas aeruginosa* (13.14%), and *Comamonas testosteroni* (5.41%). *E. coli* showed high resistance to ampicillin (84.56%), trimethoprim-sulfamethoxazole (55.67%), and ceftriaxone (48.85%), while resistance to piperacillin/tazobactam and imipenem was low (0.19% and 0.67%). ESBL-positive *E. coli* rates were similar across the 1–5, 6–11, and 12–17 years age groups (45.98%, 47.81%, and 47.64%, respectively; *P* = 0.910), and no significant age-group differences were observed for the selected resistance outcomes. *P. aeruginosa* resistance to all tested agents was ≤2%. *C. testosteroni* exhibited high resistance to ciprofloxacin (72.82%) but remained susceptible to piperacillin/tazobactam and imipenem. In pathology-stratified cases (*n* = 1,311), polymicrobial infection was more common in gangrenous than non-gangrenous appendicitis (42.07% vs. 33.70%, *P* = 0.046), and *P. aeruginosa* was detected more frequently (26.21% vs. 17.50%, *P* = 0.011).

**Conclusion:**

In Ningbo, *E. coli* was the predominant bacterial pathogen isolated from intraoperative specimens collected from children with acute appendicitis, often co-occurring with *P. aeruginosa*; *C. testosteroni* ranked the third. Substantial resistance in *E. coli* suggested that empiric reliance on third-generation cephalosporins may be insufficient, while age stratification did not identify any age group of children with significantly higher *E. coli* resistance. Broader Gram-negative coverage may be warranted for gangrenous disease.

## 1 Introduction

Acute appendicitis is a common cause of acute abdomen in children and often has a more abrupt onset than in adults. Luminal obstruction of the appendix and subsequent bacterial infection are considered key mechanisms in the development of acute appendicitis. According to the pathological process of inflammatory progression, acute appendicitis can be classified as acute simple appendicitis, acute suppurative appendicitis, acute gangrenous/perforated appendicitis, and periappendiceal abscess. In this context, acute simple appendicitis refers to early inflammation confined mainly to the mucosa and submucosa; acute suppurative appendicitis is characterized by transmural neutrophilic inflammation and purulent exudation; acute gangrenous/perforated appendicitis indicates transmural necrosis with or without perforation; and periappendiceal abscess refers to a localized abscess adjacent to the inflamed or perforated appendix (1–3). Once diagnosed, appendectomy is the mainstay of treatment. However, when a periappendiceal abscess has formed, management tends to favor nonoperative strategies, relying primarily on antimicrobial therapy with or without catheter drainage, and the optimal treatment pathway remains controversial (2).

Appendicitis is frequently accompanied by bacterial infection; therefore, effective anti-infective therapy is crucial for improving cure rates and reducing complications. Given that children are in a stage of growth and development, certain antibiotics should be used with caution. Analyzing the distribution of pathogens and their antimicrobial resistance profiles can facilitate rational antibiotic selection and effective treatment (4–6). Multiple studies both in China and abroad have shown that *Enterobacteriales* are the predominant pathogens isolated in children with acute appendicitis, with *Escherichia coli* being the most frequently detected, and antimicrobial resistance becoming increasingly severe (4, 7–11). Ningbo is a coastal port city in eastern Zhejiang Province on the southern flank of the Yangtze River Delta, with a land area of approximately 9,816 km^2^ and a 2024 permanent resident population of approximately 9.77 million, corresponding to a population density of approximately 996 persons/km^2^. In view of the serious situation of antimicrobial resistance and the lack of corresponding local data, this study retrospectively reviewed the distribution of pathogens and antimicrobial susceptibility patterns of intraoperative specimens from children with acute appendicitis in the Ningbo region from 2019 to 2024, aiming to provide more targeted evidence for local empiric antimicrobial therapy.

## 2 Methods

### 2.1 Study design and participants

This retrospective study included children aged below 18 years who underwent appendectomy at a tertiary women's and children's hospital in Ningbo, China between January 2019 and December 2024 and had a postoperative pathological diagnosis of acute appendicitis. The study was reviewed and approved by the institutional ethics committee.

### 2.2 Inclusion and exclusion criteria

#### 2.2.1 Inclusion criteria

1. Age < 18 years.
2. Clinical diagnosis of appendicitis supported by imaging and treated with appendectomy.
3. Intraoperative collection of pus, peritoneal fluid, or intra-abdominal drainage fluid for bacterial culture and susceptibility testing.
4. Pathological confirmation of acute appendicitis.

#### 2.2.2 Exclusion criteria

1. Non-operative management or previous appendectomy.
2. Specimens not submitted or unsuitable for microbiological testing due to inadequate volume or contamination.
3. Chronic appendicitis.
4. Concomitant Meckel's diverticulum, intestinal tumors, or other intra-abdominal procedures requiring simultaneous surgery.

### 2.3 Preoperative antimicrobial management

In our hospital, metronidazole combined with cephalosporins may be used as part of preoperative antimicrobial management in children with clinically diagnosed or highly suspected acute appendicitis, depending on disease severity and the treating physician's judgment.

### 2.4 Specimen collection and microbiological methods

Intraoperative specimens (pus, peritoneal fluid, or drainage fluid) were submitted to the microbiology laboratory for aerobic culture and susceptibility testing. Specimens were inoculated onto Columbia blood agar plates (Zhejiang Qinggu Biological Engineering Co., Ltd., Hangzhou, China; specification/model: 2 × 10 plates, 90 mm per plate) and incubated at 35 °C for 18–24 h. Bacterial identification was performed using a matrix-assisted laser desorption/ionization time-of-flight mass spectrometry system (model: VITEK® MS; bioMérieux, Marcy-l'Étoile, France). Antimicrobial susceptibility testing was performed using an automated system (model: VITEK 2 COMPACT; bioMérieux, Marcy-l'Étoile, France) with the manufacturer's antimicrobial susceptibility testing cards for minimum inhibitory concentration (MIC) determination. When necessary, the Kirby-Bauer disk diffusion method was used as a supplementary test using antimicrobial susceptibility discs (Oxoid Limited, Basingstoke, United Kingdom; Oxoid CT series, with catalog numbers varying according to the antimicrobial agent tested). Results were interpreted according to the Clinical and Laboratory Standards Institute M100 standard applicable to each study year (CLSI, Malvern, PA, United States). Quality control strains included *E. coli* ATCC 25922, *P. aeruginosa* ATCC 27853, *Staphylococcus aureus* ATCC 25923, and *Enterococcus faecalis* ATCC 29212.

### 2.5 Definitions and grouping

Culture positivity was defined as growth of bacterial pathogens from intraoperative specimens. For subgroup analyses, culture-positive cases with definitive pathological classification were grouped into non-gangrenous and gangrenous appendicitis. Polymicrobial infection was defined as isolation of two or more distinct bacterial species from a single specimen.

### 2.6 Statistical analysis

Statistical analyses were performed using IBM SPSS Statistics, version 29.0 (IBM Corp., Armonk, NY, United States). Categorical variables were compared using the chi-square test. Continuous variables were analyzed using the Mann–Whitney U test or independent-samples t-test depending on distribution. All tests were two-sided, and *P* < 0.05 was considered statistically significant.

## 3 Results

### 3.1 Patient characteristics

A total of 1,981 children underwent appendectomy, including six open and 1,975 laparoscopic procedures. Intraoperative cultures were positive in 1,393 cases (70.31%). Among culture-positive cases, 846 were boys and 547 were girls; age ranged from 1 to 17 years (mean 8 years 8 months). There were 253 children aged 1–5 years (18.16%), 868 aged 6–11 years (62.31%), and 272 aged 12–17 years (19.52%).

### 3.2 Pathogen distribution

In 1,393 culture-positive specimens, 1,903 isolates were recovered (Table 1). Gram-negative organisms accounted for 1,544/1,903 (81.14%), and Gram-positive organisms for 359/1,903 (18.86%). Among Gram-negative isolates, the top three were *E. coli* (1,049; 55.12%), *P. aeruginosa* (250; 13.14%), and *C. testosteroni* (103; 5.41%). Among Gram-positive isolates, the top three were *Streptococcus anginosus* (113; 5.94%), *Enterococcus avium* (71; 3.73%), and *Streptococcus constellatus* (63; 3.31%). Single-organism infection occurred in 915/1,393 cases (65.69%), with *E. coli* accounting for 670/915 (73.22%). Two-organism infection occurred in 446/1,393 cases (32.02%), of which 351/446 (78.70%) involved *E. coli* plus another organism. Three-organism infection occurred in 32 cases, most commonly involving *E. coli*, *P. aeruginosa*, and *Streptococcus anginosus* (7; 21.88%).

**Table 1:** Distribution of 1,903 bacterial isolates recovered from intraoperative specimens of 1,393 culture-positive children with acute appendicitis at a tertiary hospital in Ningbo, China, 2019–2024.

Category	Organism	Number	Percentage (%)
Gram-negative	*Escherichia coli*	1,049	55.12
	*Pseudomonas aeruginosa*	250	13.14
	*Comamonas testosteroni*	103	5.41
	*Klebsiella pneumoniae*	31	1.63
	*Cupriavidus pauculus*	18	0.95
	*Proteus mirabilis*	12	0.63
	*Aeromonas caviae*	9	0.47
	*Klebsiella oxytoca*	7	0.37
	*Proteus penneri*	7	0.37
	*Citrobacter koseri (diversus)*	5	0.26
	Other Gram-negative bacteria	53	2.79
Gram-positive	*Streptococcus anginosus*	113	5.94
	*Enterococcus avium*	71	3.73
	*Streptococcus constellatus*	63	3.31
	*Enterococcus faecalis*	19	1.00
	*Enterococcus faecium*	18	0.95
	*Enterococcus gallinarum*	10	0.53
	*Streptococcus gordonii*	8	0.42
	*Streptococcus thoraltensis*	7	0.37
	*Streptococcus gallolyticus*	6	0.32
	*Streptococcus sanguinis*	5	0.26
	Other Gram-positive bacteria	39	2.05
Total		1,903	100

### 3.3 Antimicrobial susceptibility of major Gram-negative pathogens

Susceptibility testing for the three major Gram-negative pathogens is summarized in Table 2. *E. coli* exhibited the highest resistance to ampicillin (84.56%) and substantial resistance to ampicillin/sulbactam (45.85%), trimethoprim-sulfamethoxazole (55.67%), and ceftriaxone (48.85%). Resistance to aztreonam, gentamicin, and tobramycin was 26.15%, 30.60%, and 6.77%, respectively, and resistance to ceftazidime was 14.20%. Resistance to piperacillin/tazobactam and imipenem remained low (0.19% and 0.67%). The proportion of extended-spectrum *β*-lactamase (ESBL)-producing *E. coli* was 47.47%. *P. aeruginosa* showed low resistance to all tested agents (≤2%), with no resistance detected to piperacillin/tazobactam and an imipenem resistance rate of 0.40%. *C. testosteroni* demonstrated marked resistance to ciprofloxacin (72.82%) and aztreonam (47.57%), moderate resistance to trimethoprim-sulfamethoxazole (33.01%) and ceftazidime (22.33%), but remained fully susceptible to piperacillin/tazobactam and imipenem.

**Table 2:** Number and percentage of resistant isolates among the three predominant Gram-negative pathogens recovered from intraoperative specimens of children with acute appendicitis at a tertiary hospital in Ningbo, China, 2019–2024.

Antibiotic	*E. coli* (*n* = 1,049)	*P. aeruginosa* (*n* = 250)	*C. testosteroni* (*n* = 103)
Ampicillin	887 (84.56%)	–	–
Ampicillin/sulbactam	481 (45.85%)	–	–
Piperacillin/tazobactam	2 (0.19%)	0 (0%)	0 (0%)
Ceftazidime	149 (14.20%)	2 (0.80%)	23 (22.33%)
Ceftriaxone	512 (48.85%)	–	1 (0.97%)
Aztreonam	274 (26.15%)	NR	49 (47.57%)
Imipenem	7 (0.67%)	1 (0.40%)	0 (0%)
Gentamicin	321 (30.60%)	2 (0.80%)	12 (11.65%)
Tobramycin	88 (6.77%)	2 (0.80%)	12 (11.65%)
Ciprofloxacin	456 (43.51%)	5 (2.00%)	75 (72.82%)
Trimethoprim–sulfamethoxazole	584 (55.67%)	–	34 (33.01%)

Values are number of resistant isolates (resistance rate). “-” indicates that susceptibility testing was not performed for that organism-agent combination. NR indicates that results were not reported due to insufficient data (e.g., very small number tested and/or substantial missing values), to avoid unstable estimates. Resistance rates were calculated using the number of isolates actually tested as the denominator.

### 3.4 Age-stratified antimicrobial resistance in *E. coli*

Among 1,049 *E. coli* isolates, age-stratified analysis included 175 isolates from children aged 1–5 years, 662 from those aged 6–11 years, and 212 from those aged 12–17 years (Table 3). ESBL-positive rates were 45.98%, 47.81%, and 47.64%, respectively (*P* = 0.910). Resistance to ampicillin/sulbactam (50.29%, 45.29%, and 45.02%; *P* = 0.471), ceftazidime (11.43%, 14.13%, and 17.06%; *P* = 0.285), ceftriaxone (48.00%, 49.09%, and 48.82%; *P* = 0.967), trimethoprim-sulfamethoxazole (58.29%, 56.80%, and 50.00%; *P* = 0.166), and ciprofloxacin (38.86%, 45.02%, and 42.65%; *P* = 0.330) did not differ significantly across age groups.

**Table 3:** Age-stratified antimicrobial resistance of *Escherichia coli* isolates recovered from intraoperative specimens of children with acute appendicitis at a tertiary hospital in Ningbo, China, 2019–2024.

Resistance outcome	1–5 years (*n* = 175)	6–11 years (*n* = 662)	12–17 years (*n* = 212)	*P*-value
ESBL-producing *E. coli*	80 (45.98%)	316 (47.81%)	101 (47.64%)	0.910
Ampicillin/sulbactam	88 (50.29%)	298 (45.29%)	95 (45.02%)	0.471
Ceftazidime	20 (11.43%)	93 (14.13%)	36 (17.06%)	0.285
Ceftriaxone	84 (48.00%)	325 (49.09%)	103 (48.82%)	0.967
Trimethoprim-sulfamethoxazole	102 (58.29%)	376 (56.80%)	106 (50.00%)	0.166
Ciprofloxacin	68 (38.86%)	298 (45.02%)	90 (42.65%)	0.330

Values are number of resistant or ESBL-producing isolates (percentage). Percentages were calculated using the number of isolates tested for each outcome as the denominator. ESBL, extended-spectrum beta-lactamase.

### 3.5 Pathogen distribution by pathological subtype

Of the 1,393 culture-positive cases, 82 pathology reports stated only “acute appendicitis” without further classification and were excluded from pathological stratification. The remaining 1,311 cases were classified as non-gangrenous (*n* = 1,166) or gangrenous appendicitis (*n* = 145). Polymicrobial infection was more frequent in gangrenous than non-gangrenous appendicitis (42.07% vs. 33.70%, *P* = 0.046; Table 4).

**Table 4:** Monomicrobial and polymicrobial infections among 1,311 culture-positive children with acute appendicitis, stratified by non-gangrenous and gangrenous pathological subtype, at a tertiary hospital in Ningbo, China, 2019–2024.

Infection type	Non-gangrenous (*n* = 1,166)	Gangrenous (*n* = 145)	*P*-value
Monomicrobial (one organism), *n* (%)	773 (66.30%)	84 (57.93%)	0.046
Polymicrobial (two or more organisms), *n* (%)	393 (33.70%)	61 (42.07%)	

Values are number of children (percentage). Percentages were calculated within each pathological subtype.

Major pathogen distributions are shown in Table 5. *E. coli* predominated in both groups (75.81% vs. 73.79%, *P* = 0.593). *P. aeruginosa* was detected significantly more often in gangrenous appendicitis (26.21% vs. 17.50%, *P* = 0.011). In contrast, *Streptococcus constellatus* was more frequent in non-gangrenous appendicitis (5.06% vs. 0.69%, *P* = 0.018).

**Table 5:** Detection of major pathogens among 1,311 culture-positive children with acute appendicitis, stratified by non-gangrenous and gangrenous pathological subtype, at a tertiary hospital in Ningbo, China, 2019–2024.

Pathogen, *n* (%)	Non-gangrenous (*n* = 1,166)	Gangrenous (*n* = 145)	*P*-value
*Escherichia coli*	884 (75.81%)	107 (73.79%)	0.593
*Pseudomonas aeruginosa*	204 (17.50%)	38 (26.21%)	0.011
*Comamonas testosteroni*	83 (7.12%)	14 (9.66%)	0.271
*Streptococcus anginosus*	100 (8.58%)	9 (6.21%)	0.330
*Enterococcus avium*	55 (4.72%)	10 (6.90%)	0.246
*Streptococcus constellatus*	59 (5.06%)	1 (0.69%)	0.018

Values are number of children in whom the pathogen was detected (percentage within the pathological subtype). Because multiple pathogens could be isolated from one child, percentages do not sum to 100%.

## 4 Discussion

In this retrospective analysis of intraoperative cultures from children undergoing appendectomy, the overall culture positivity rate was 70.31%, comparable to previous reports (4, 9, 12). Gram-negative bacteria predominated (81.14%), consistent with the classic concept that *Enterobacteriales* are the main pathogens in appendicitis (12, 13). *E. coli* was the most frequently isolated organism (55.12%). Notably, *P. aeruginosa* ranked second (13.14%), aligning with several studies of appendicitis in children (4, 14). However, *C. testosteroni* ranked third among Gram-negative pathogens (5.41%), whereas it has been uncommon in prior appendicitis series (4, 15). This discrepancy may reflect geographic and environmental factors; Ningbo is a coastal city, and local water and environmental microbiota may increase exposure to *Comamonas* species (16). These findings suggest that while appendicitis pathogens are largely gut-derived, regional variation exists and should be considered when designing empiric regimens.

Resistance profiling showed that *E. coli* had high resistance to ampicillin, ampicillin/sulbactam, trimethoprim-sulfamethoxazole, and ceftriaxone, broadly consistent with national surveillance data from China (11). Such resistance may compromise empiric regimens that rely on third-generation cephalosporins. Fluoroquinolone resistance was also substantial, and quinolones may affect cartilage development in children, limiting their routine use (17, 18). Although tobramycin resistance was low, potential ototoxicity and nephrotoxicity require careful risk-benefit assessment (19, 20). The high resistance burden in *E. coli* is likely driven by ESBL production, which can inactivate penicillins, cephalosporins, and monobactams and is often associated with multidrug resistance (21). Encouragingly, *E. coli* remained highly susceptible to piperacillin/tazobactam and imipenem, supporting these agents as reliable options for severe infection when indicated. *P. aeruginosa* exhibited very low resistance (≤2%) in our cohort, lower than the corresponding CHINET surveillance estimate from China (11). This may relate to infection type and pathogen sources, but ongoing monitoring is warranted, especially for carbapenem-resistant strains. *C. testosteroni* demonstrated a multidrug-resistant phenotype, with very high ciprofloxacin resistance, which is compatible with the organism's capacity to produce *β*-lactamases and acquire resistance determinants (16, 22). Despite this, it remained fully susceptible to piperacillin/tazobactam and imipenem in our dataset. In addition, the detection of Gram-positive bacteria in this study was mainly streptococci and enterococci, which is consistent with previous studies (6, 15), and no linezolid-resistant strains were found in either genus.

The age-stratified *E. coli* analysis did not show significant differences in ESBL positivity or resistance to third-generation cephalosporins, ampicillin/sulbactam, trimethoprim-sulfamethoxazole, or ciprofloxacin across the three age groups of children. These findings suggest that the observed *E. coli* resistance burden is broadly distributed across childhood rather than concentrated in a single age stratum. Empiric therapy decisions in this cohort should therefore be guided primarily by local susceptibility patterns and disease severity, while individual age-related drug safety considerations remain important.

We further observed that as disease severity increased to gangrenous appendicitis, polymicrobial infection became more common, consistent with prior studies (4, 15, 23). Complex appendicitis often involves mixed aerobic–anaerobic infection (7, 15, 24). Because anaerobes are typically susceptible to metronidazole or ornidazole and these agents are routinely included in empiric regimens (2, 4), anaerobic culture and susceptibility testing are not routinely performed in our setting. Importantly, *P. aeruginosa* was detected more frequently in gangrenous appendicitis (26.21% vs. 17.50%), suggesting an association with more severe disease and greater microbiological complexity (13, 25). Therefore, stratifying empiric therapy by pathological severity may be reasonable: for suspected complicated or gangrenous appendicitis, a *β*-lactam/*β*-lactamase inhibitor with good Gram-negative coverage may be preferred, with early de-escalation once culture results are available, and antipseudomonal coverage may be particularly relevant for gangrenous disease.

This study has limitations. First, it was a single-center retrospective study, and results may not generalize to other regions. Second, anaerobic pathogens were not systematically cultured, which may underestimate polymicrobial infection. Third, clinical outcomes and prior antibiotic exposure were not analyzed and should be addressed in future work.

## 5 Conclusion

In intraoperative specimens from children with acute appendicitis in Ningbo, *E. coli* was predominant and often co-occurred with *P. aeruginosa*; *C. testosteroni* ranked the third. *E. coli* demonstrated high resistance to ampicillin and third-generation cephalosporins, which may limit empiric use of these agents. Age-stratified *E. coli* analysis showed no significant differences in ESBL positivity or selected resistance outcomes across age groups of children. Gangrenous appendicitis was associated with more polymicrobial infection and higher detection of *P. aeruginosa*, supporting severity-stratified empiric therapy and continued local resistance surveillance.

## Data Availability

The original contributions presented in the study are included in the article/Supplementary Material, further inquiries can be directed to the corresponding author/s.

## References

[1] Carr NJ. The pathology of acute appendicitis. Ann Diagn Pathol. (2000) 4:46–58. 10.1016/S1092-9134(00)90011-X 10684382

[2] Di Saverio S Podda M De Simone B Ceresoli M Augustin G Gori A. Diagnosis and treatment of acute appendicitis: 2020 update of the WSES Jerusalem guidelines. World J Emerg Surg. (2020) 15:27. 10.1186/s13017-020-00306-3 32295644 PMC7386163

[3] Salminen P Haijanen J Minneci PC Davidson GH Boermeester MA Livingston E. Appendicitis. Nat Rev Dis Primers. (2025) 11:79. 10.1038/s41572-025-00659-6 41233355

[4] Felber J Gross B Rahrisch A Waltersbacher E Trips E Schröttner P. Bacterial pathogens in pediatric appendicitis: a comprehensive retrospective study. Front Cell Infect Microbiol. (2023) 13:1027769. 10.3389/fcimb.2023.1027769 37228669 PMC10205019

[5] Di Mitri M Collautti E Thomas E Di Carmine A Veronesi G Cravano SM. The care of appendicular peritonitis in the era of antibiotic resistance: the role of surgery and the appropriate antibiotic choice. Gastrointest Disord. (2024) 6:964–75. 10.3390/gidisord6040067

[6] Aiyoshi T Masumoto K Tanaka N Sasaki T Chiba F Ono K. Optimal first-line antibiotic treatment for pediatric complicated appendicitis based on peritoneal fluid culture. Pediatr Gastroenterol Hepatol Nutr. (2021) 24:510–7. 10.5223/pghn.2021.24.6.510 34796095 PMC8593360

[7] Yu CH Chang CN Wang CC. Causative microbes and antibiotic susceptibility of acute appendicitis in adults and children. Pediatr Neonatol. (2024) 65:159–64. 10.1016/j.pedneo.2023.08.003 37741758

[8] Melamed R Ozalvo D Sagi O Assi Z Nahom A Kezerle Y. Rising multidrug-resistant pathogens in pediatric appendicitis: a decade-long study from southern Israel. Eur J Pediatr Surg. (2025) 35:375–81. 10.1055/a-2540-3690 40148128

[9] Brits E Kruger E Fivaz K Oosthuizen K Joubert M van Pletzen P-M. Type and antibiotic susceptibility of bacteria cultured in paediatric acute appendicitis. S Afr J Infect Dis. (2025) 40:689. 10.4102/sajid.v40i1.689 40060088 PMC11886553

[10] World Health Organization. Global antimicrobial resistance and use surveillance system (GLASS) report 2022. (2022). Available online at: https://www.who.int/publications/i/item/9789240062702 (Accessed June 28, 2026).

[11] Guo Y Hu FP Zhu DM Wang F Jiang XF Xu YC. Antimicrobial resistance profile of clinical isolates in hospitals across China: report from the CHINET antimicrobial resistance surveillance program, 2023. Chin J Infect Chemother. (2024) 24:627–37 (in Chinese). 10.16718/j.1009-7708.2024.06.001

[12] Naji H Jayakumar J Ali R. Bacterial profile and antibiotic selection for pediatric appendicitis: a retrospective cohort study. Surg Open Sci. (2023) 14:120–3. 10.1016/j.sopen.2023.07.018 37554312 PMC10404798

[13] Bhaskar K Clarke S Moore LSP Hughes S. Bacterial peritonitis in paediatric appendicitis; microbial epidemiology and antimicrobial management. Ann Clin Microbiol Antimicrob. (2023) 22:45. 10.1186/s12941-023-00591-1 37270568 PMC10239121

[14] Garzon-González LN Padilla LT Patiño F Hernández MA Valero J Molina ID. Association between bacterial resistance profile and the development of intra-abdominal abscesses in pediatric patients with perforated appendicitis: cohort study. Pediatr Surg Int. (2023) 40:18. 10.1007/s00383-023-05570-3 38082019 PMC10713695

[15] Zachos K Kolonitsiou F Panagidis A Gkentzi D Fouzas S Alexopoulos V. Association of the bacteria of the vermiform appendix and the peritoneal cavity with complicated acute appendicitis in children. Diagnostics. (2023) 13:1839. 10.3390/diagnostics13111839 37296691 PMC10252422

[16] Ryan MP Sevjahova L Gorman R White S. The emergence of the genus Comamonas as important opportunistic pathogens. Pathogens. (2022) 11:1032. 10.3390/pathogens11091032 36145464 PMC9504711

[17] Li Y Wang J Wang CL Chen L. Safety analysis of quinolones use in minors—based on the FAERS database. Front Med. (2024) 11:1437376. 10.3389/fmed.2024.1437376 PMC11390674 39267976

[18] Wang JG Cui HR Hu YS Tang HB. Assessment of the risk of musculoskeletal adverse events associated with fluoroquinolone use in children: a meta-analysis. Medicine (Baltimore). (2020) 99:e21860. 10.1097/MD.0000000000021860 32846837 PMC7447478

[19] Rivetti S Romano A Mastrangelo S Attinà G Maurizi P Ruggiero A. Aminoglycosides-related ototoxicity: mechanisms, risk factors, and prevention in pediatric patients. Pharmaceuticals. (2023) 16:1353. 10.3390/ph16101353 37895824 PMC10610175

[20] Thy M Timsit JF de Montmollin E. Aminoglycosides for the treatment of severe infection due to resistant Gram-negative pathogens. Antibiotics. (2023) 12:860. 10.3390/antibiotics12050860 37237763 PMC10215420

[21] Rodríguez-Baño J Gutiérrez-Gutiérrez B Machuca I Pascual A. Treatment of infections caused by extended-spectrum-beta-lactamase-, AmpC-, and carbapenemase-producing Enterobacteriaceae. Clin Microbiol Rev. (2018) 31:e00079–17. 10.1128/CMR.00079-17 29444952 PMC5967687

[22] Li Y Fang C Wang X Liu Q Qiu Y Dai X. A new class A beta-lactamase gene *bla*_CAE−1_ coexists with *bla*_AFM−1_ in a novel untypable plasmid in Comamonas aquatica. Sci Rep. (2023) 13:3634. 10.1038/s41598-023-28312-w 36869066 PMC9984417

[23] Plattner AS Newland JG Wallendorf MJ Shakhsheer BA. Management and microbiology of perforated appendicitis in pediatric patients: a 5-year retrospective study. Infect Dis Ther. (2021) 10:2247–57. 10.1007/s40121-021-00502-x 34287780 PMC8572942

[24] Kakar M Reinis A Kroica J Engelis A Broks R Asare L. Microbiota assessment of pediatric simple and complex acute appendicitis. Medicina (B Aires). (2022) 58:1144. 10.3390/medicina58091144 PMC9500912 36143821

[25] Theodorou CM Stokes SC Hegazi MS Brown EG Saadai P. Is Pseudomonas infection associated with worse outcomes in pediatric perforated appendicitis? J Pediatr Surg. (2021) 56:1826–30. 10.1016/j.jpedsurg.2020.10.031 33223225 PMC8096855

